# The value of genomic relationship matrices to estimate levels of inbreeding

**DOI:** 10.1186/s12711-021-00635-0

**Published:** 2021-05-01

**Authors:** Beatriz Villanueva, Almudena Fernández, María Saura, Armando Caballero, Jesús Fernández, Elisabeth Morales-González, Miguel A. Toro, Ricardo Pong-Wong

**Affiliations:** 1 Departamento de Mejora Genética Animal, INIA, Ctra. de La Coruña, km 7.5, 28040 Madrid, Spain; 2 Centro de Investigación Mariña, Universidade de Vigo, Departamento de Bioquímica, Genética E Inmunología, Campus de Vigo, 36310 Vigo, Spain; 3 Departamento de Producción Agraria, ETSI Agrónomos, Universidad Politécnica de Madrid, 28040 Madrid, Spain; 4 Genetics and Genomics, The Roslin Institute and the R(D)SVS, University of Edinburgh, Easter Bush, Midlothian, EH25 9RG UK

## Abstract

**Background:**

Genomic relationship matrices are used to obtain genomic inbreeding coefficients. However, there are several methodologies to compute these matrices and there is still an unresolved debate on which one provides the best estimate of inbreeding. In this study, we investigated measures of inbreeding obtained from five genomic matrices, including the Nejati-Javaremi allelic relationship matrix (*F*_*NEJ*_), the Li and Horvitz matrix based on excess of homozygosity (*F*_*L&H*_), and the VanRaden (methods 1, *F*_*VR*1_, and 2, *F*_*VR*2_) and Yang (*F*_*YAN*_) genomic relationship matrices. We derived expectations for each inbreeding coefficient, assuming a single locus model, and used these expectations to explain the patterns of the coefficients that were computed from thousands of single nucleotide polymorphism genotypes in a population of Iberian pigs.

**Results:**

Except for *F*_*NEJ*_, the evaluated measures of inbreeding do not match with the original definitions of inbreeding coefficient of Wright (correlation) or Malécot (probability). When inbreeding coefficients are interpreted as indicators of variability (heterozygosity) that was gained or lost relative to a base population, both *F*_*NEJ*_ and *F*_*L&H*_ led to sensible results but this was not the case for *F*_*VR*1_, *F*_*VR*2_ and *F*_*YAN*_. When variability has increased relative to the base, *F*_*VR*1_, *F*_*VR*2_ and *F*_*YAN*_ can indicate that it decreased. In fact, based on *F*_*YAN*_, variability is not expected to increase. When variability has decreased, *F*_*VR*1_ and *F*_*VR*2_ can indicate that it has increased. Finally, these three coefficients can indicate that more variability than that present in the base population can be lost, which is also unreasonable. The patterns for these coefficients observed in the pig population were very different, following the derived expectations. As a consequence, the rate of inbreeding depression estimated based on these inbreeding coefficients differed not only in magnitude but also in sign.

**Conclusions:**

Genomic inbreeding coefficients obtained from the diagonal elements of genomic matrices can lead to inconsistent results in terms of gain and loss of genetic variability and inbreeding depression estimates, and thus to misleading interpretations. Although these matrices have proven to be very efficient in increasing the accuracy of genomic predictions, they do not always provide a useful measure of inbreeding.

**Supplementary Information:**

The online version contains supplementary material available at 10.1186/s12711-021-00635-0.

## Background

Inbreeding, i.e. the mating of individuals related by ancestry, is a fundamental concept in many areas of biology, including animal and plant breeding [1], human genetics [2, 3], and evolutionary [4] and conservation biology [5]. Inbreeding results in a reduction of genetic diversity, as it increases homozygosity at the expense of heterozygosity. This increase in homozygosity in turn increases the incidence of homozygous recessive defects and decreases population means for many quantitative traits (i.e., inbreeding depression), particularly those related to fitness [6, 7].

The level of inbreeding of an individual is measured by the inbreeding coefficient, which was defined by Wright as the correlation between homologous alleles of the two gametes that unite to form the individual [8], and later by Malécot as the probability that two homologous alleles at a given locus are identical-by-descent [9]. The inbreeding coefficient also gives the proportion by which the heterozygosity of an individual is reduced by inbreeding [10] and, thus, the proportional loss of genetic variation. Classically, the inbreeding coefficient of an individual has been determined based on its pedigree. However, the pedigree-based inbreeding coefficient provides only expected proportions of the genome that are identical-by-descent.

The level of inbreeding has also been estimated from molecular data, such as those contained in high-density single nucleotide polymorphism (SNP) arrays. Genomic inbreeding coefficients can be more accurate than pedigree-based measures because they capture the variation due to Mendelian sampling and therefore can differentiate among individuals with the same pedigree (e.g. [11]). Genomic measures also allow us to differentiate inbreeding at specific regions of a genome, which is not possible with pedigree-based inbreeding.

Several methods have been proposed to calculate inbreeding coefficients using genomic data, including methods based on continuous runs of homozygosity (e.g. [11, 12] and methods applied on a SNP-by-SNP basis (e.g. [13–16]). Some of the latter measures come from matrices that are used to obtain genomic predictions in animal breeding. In this context, best linear unbiased predicted (BLUP) evaluations are replaced by genomic BLUP (GBLUP) evaluations, in which the numerator relationship matrix (NRM) is substituted by one of several genomic relationship matrices (GRM) [15, 16]. Given that the diagonals of the NRM equal 1 plus the inbreeding coefficients for the corresponding individuals, it has been generally accepted that the diagonals of the GRM are 1 plus the realized inbreeding level for the corresponding individuals. These genomic measures of inbreeding have been widely used [11, 17–47]. However, they can result in very different outcomes and the correlations between these estimators vary greatly and can even be negative, e.g. [27, 35]. Thus, there is still an unresolved debate on which are the best measures of inbreeding.

In this study, we compared genomic inbreeding coefficients that were obtained from different SNP-by-SNP methods to understand their relationship with traditional definitions of inbreeding. First, we describe different coefficients based on genomic information at the individual level. Second, we derive expectations at the population level for the different coefficients based on a single locus model. These expectations are then used to explain the patterns of the coefficients computed based on thousands of SNP genotypes across the genome in a highly inbred pig population.

## Methods

### Inbreeding coefficients obtained from genomic data

Individual inbreeding coefficients were obtained from the diagonal elements of five different genomic relationship matrices. These coefficients have been widely used in the literature, but under different names (see Table 1) and there is no consensus about the nomenclature. Here, the name chosen for each coefficient makes reference to the authors who first proposed or formulated it explicitly, to the best of our knowledge. We compared the following coefficients:

**Table 1:** Summary of the names given to different genomic inbreeding coefficients in the literature

Nomenclature used in this paper	Nomenclature used in the literature	References
*F*_*NEJ*_	*F*_PH_	[19]
	*F*_M_	[20]
	*F*_MOL_	[33]
	Homozygosity	[21]
	*F*_HOM_	[28, 35]
	HOM_SNP_	[37]
	SNP-Similarity*	[29]
	SIM*	[47]
*F*_*L&H*_	*F*_h_ or *F*_H_	[11, 25, 40]
	*F*_snp_	[26]
	*F*_HOM_	[27, 33, 36, 41, 42, 46, 55]
	*F*_ExHOM_	[35]
	*F*_PLINK_	[31]
	*F*_IS_	[45]
	*F*_EH_	[34]
	LHR	[22]
	L&H*	[47]
*F*_*VR*1_	*F*_GRM_	[19, 41]
	*F*_GRM1_	[35]
	*F*_VR_	[34]
	*F*_G_	[17]
	VR1*	[47]
*F*_*VR*2_	*F*hatI, *F*^*I*^	[18, 42, 54]
	*F*_GRM_	[27, 33]
	F_GRM2_	[35]
	VR2*	[47]
*F*_*YAN*_	*F*hatIII, *F*^*III*^	[18, 42, 54]
	*F*_alt_	[11, 40]
	GRM_F, *F*_GRM_	[21, 28, 31]
	*F*_UNI_	[27, 35, 36, 41, 55]
	*F*_grm_	[31]
	SNP-Yang*	[29]
	YAN*	[47]

^*^Self-relationship or self-coancestry

1. ${F}_{NEJ}$: inbreeding coefficient computed from the diagonal elements of the allelic relationship matrix of Nejati-Javaremi et al. [14] as:
  $${F}_{NEJ}= \frac{\sum_{k=1}^{S}(\sum_{i=1}^{2}\sum_{j=1}^{2}{I}_{{ij}_{k}})/2}{S}-1,$$
  where ${I}_{{ij}_{k}}$ is the identity of the two alleles ($i$ and $j$) of the individual at SNP $k$, which takes the value of 1 if the two alleles are identical and 0 if they are not. Note that ${F}_{NEJ}$ is simply the proportion of SNPs that are homozygous for the individual and thus it does not distinguish between identity-by-state (IBS) and identity-by-descent (IBD) [48].
2. ${F}_{L\&H}$: inbreeding coefficient based on the relationship matrix that describes deviations from Hardy–Weinberg proportions, computed as:
  $${F}_{L\&H}=\frac{S{F}_{NEJ}-\sum_{k=1}^{S}[1-2{p}_{k\left(0\right)}(1-{p}_{k\left(0\right)})]}{S-\sum_{k=1}^{S}[1-2{p}_{k\left(0\right)}(1-{p}_{k\left(0\right)})]},$$
  where ${p}_{k(0)}$ is the frequency of the reference allele (allele *B*) of SNP $k$ in the base (reference) population [13]. ${F}_{L\&H}$ estimates the deviation of the observed frequency of homozygotes (*AA* and *BB*) from that expected in the base population under Hardy–Weinberg proportions. Thus, it corrects for the homozygosity that was present in the base population and expresses molecular inbreeding in terms of IBD [42, 48, 49].
3. *F*_*VR*1_: inbreeding coefficient computed from the diagonal elements of the genomic relationship matrix obtained according to VanRaden’s method 1 [15], as follows:
  $${F}_{VR1}=\frac{\sum_{k=1}^{S}{({x}_{k}-{2p}_{k(0)})}^{2}}{2\sum_{k=1}^{S}{p}_{k(0)}(1-{p}_{k(0)})}-1,$$
  where ${x}_{k}$ is the genotype of the individual for SNP $k$, coded as 0, 1 or 2 for genotypes *AA*, *AB* and *BB*, respectively, and ${p}_{k(0)}$ is as defined for ${F}_{L\&H}$. ${F}_{VR1}$ is based on the variance of additive genetic values and provides a measure relative to frequencies of the reference allele in the base population. However, ${F}_{VR1}$ differs from *F*_*L&H*_ in that with ${F}_{VR1}$ homozygous genotypes are weighted by the inverse of their allele frequency and, thus, rare homozygous genotypes contribute more to the inbreeding measure than common homozygous genotypes [35].
4. ${F}_{VR2}$: inbreeding coefficient computed from the diagonal elements of the genomic relationship matrix obtained according to VanRaden’s method 2 [15] as follows:
  $${F}_{VR2}=\frac{1}{S}\sum_{k=1}^{S}\frac{{({x}_{k}-2{p}_{k(0)})}^{2}}{2{p}_{k(0)}(1-{p}_{k(0)})}-1,$$
  where ${x}_{k}$ and ${p}_{k(0)}$ are as for ${F}_{VR1}$. ${F}_{VR2}$ is similar to ${F}_{VR1}$ but the summation across markers is made differently, such that the weight given to rare alleles is even greater. In ${F}_{VR2}$, the contribution of each SNP is divided by its own variance, whereas in ${F}_{VR1}$ the contributions of all SNPs are divided by the same denominator [35].
5. ${F}_{YAN}$: inbreeding coefficient computed from the diagonal elements of the genomic relationship matrix of Yang [16] as follows:
  $${F}_{YAN}=\frac{1}{S}\sum_{k=1}^{S}\frac{ {x}_{k}^{2}-\left(1+2{p}_{k(0)}\right){x}_{{k}_{i}}+2{p}_{k(0)}^{2}}{2{p}_{k(0)}(1-{p}_{k(0)})},$$
  where ${x}_{k}$ and ${p}_{k(0)}$ are as for ${F}_{VR1}$. This coefficient is based on the correlation between uniting gametes [16, 42] and also gives more weight to homozygotes for the minor allele than to homozygotes for the major allele [40]. However, it has a lower sampling variance than the previous coefficients [18, 35] because it accounts for the sampling error associated with each SNP [16, 28].

The coefficients that depend on allele frequencies, i.e. ${F}_{L\&H}$, ${F}_{VR1}$, ${F}_{VR2}$, and ${F}_{YAN}$, need to be computed using the initial frequencies; i.e. those in the base population. Note that ${F}_{NEJ}$ is equivalent to ${F}_{VR1}$, ${F}_{VR2}$ and ${F}_{YAN}$ when base population allele frequencies equal 0.5 [29].

### Expected genomic inbreeding coefficients at the population level: a single locus model

Expected values for ${F}_{L\&H}$, ${F}_{VR1}$, ${F}_{VR2}$ and ${F}_{YAN}$ at the population level were derived based on a single SNP model. Let ${p}_{(0)}$ be the frequency of allele *B* in the base population. After $t$ generations, the frequency will have changed to ${p}_{(t)}$ due to random drift and selection, among other reasons. Assuming random mating, we can expect that genotype frequencies within a generation are in Hardy–Weinberg equilibrium. Thus, the expected $F$ for a group of individuals from generation $t$ can be obtained as:

$$E\left(F\right)=\left[freq\left(\mathrm{AA}\right){F}_{\mathrm{AA}}+freq\left(\mathrm{AB}\right){F}_{\mathrm{AB}}+freq\left(\mathrm{BB}\right){F}_{\mathrm{BB}}\right],$$

where $freq\left(\mathrm{AA}\right)={(1-{p}_{\left(t\right)})}^{2}$, $freq\left(\mathrm{AB}\right)=2{p}_{\left(t\right)}(1-{p}_{t(t)})$ and $freq\left(\mathrm{BB}\right)={p}_{(t)}^{2}$, and ${F}_{\mathrm{XY}}$ is the inbreeding coefficient for an individual with genotype $XY$, which is computed using the initial frequency ${p}_{\left(0\right)}$, as described in the previous section. To assess the impact of initial and current allele frequencies on expected values of the evaluated inbreeding coefficients, the latter were evaluated for the whole range of values for ${p}_{(0)}$ and ${p}_{(t)}$.

### Evaluation of genomic inbreeding in a population of Guadyerbas pigs

Results from the single locus model were evaluated in a population of Iberian pigs, with thousands of SNPs used to compute the inbreeding coefficients across the genome and at specific genomic regions.

#### Pig samples and SNP genotypes

The data used were from a herd of Guadyerbas Iberian pigs. The Guadyerbas strain is one of the most ancient surviving Iberian strains. It is highly inbred and in serious danger of extinction. The strain originated from four males and 20 females [50] and was conserved from 1944 until 2011 as a genetically isolated population. Accurate and complete genealogy was available from when the herd was first established (about 25 generations) and included 1178 animals born from 197 sires and 467 dams.

DNA samples were available for 86 males and 141 females born in the herd between 1992 and 2011 and were genotyped with the Illumina PorcineSNP60 BeadChip v1. SNP positions in the genome were based on the genome assembly Sscrofa 11.1. After quality control, as described in Saura et al. [20], 219 animals and 47,120 SNPs remained. In Iberian pigs, the generation interval is about three years, and thus for analysis of genomic inbreeding, we considered six cohorts of animals born in successive periods of three years, starting from year 1994 (Table 2).

**Table 2:** Number of genotyped animals per cohort and sex in the Guadverbas population

Cohort	Birth year range	Males	Females
1	1994–1996	13	18
2	1997–1999	10	42
3	2000–2002	24	18
4	2003–2005	8	19
5	2006–2008	8	7
6	2009–2011	19	29
Total		82	133

#### Patterns of genomic inbreeding coefficients

Genomic coefficients were obtained for all genotyped pigs using the SNPs that segregated in cohort 1 (17,951 SNPs). The frequencies used to calculate ${F}_{L\&H}$, ${F}_{VR1}$, ${F}_{VR2}$, and ${F}_{YAN}$ were those for cohort 1 (i.e. this cohort was considered to be the base population). Patterns of inbreeding across the genome were determined using sliding windows of 35 SNPs (average length of 4.25 Mb) that were moved one SNP at a time (17,339 windows). For each window, the average $F$ was computed in order to reduce the noisiness of single-locus estimates and to clarify the graphical representations [51–53]. For the coefficients that depend on allele frequencies (${F}_{L\&H}$, ${F}_{VR1}$, ${F}_{VR2}$ and ${F}_{YAN}$), the formulae were applied within each window. Finally, values were averaged across individuals.

#### Inbreeding depression

The behavior of the different genomic inbreeding coefficients will have consequences when they are used to estimate the rate of inbreeding depression across the genome. In order to investigate this, we performed a genome scan for inbreeding depression for the number of piglets born alive in the Guadyerbas population, using all genotyped sows with records born in the six cohorts (109 sows and 265 litter records) and the sliding window approach. The animals and phenotypic data used and the model fitted are described in detail in Saura et al. [26]. Briefly, inbreeding depression was estimated by regressing the number of piglets born alive on *F* assuming a linear model. Fixed effects included the combination of season of farrowing and farrowing facilities, parity, strain of boar, and the linear regression on $F$. Random effects included additive genetic, permanent environmental, and residual effects. The variance–covariance matrix of additive genetic effects was assumed to be the pedigree-based numerator relationship matrix. Three measures of $F$ (${F}_{L\&H}$, ${F}_{VR2}$ and ${F}_{YAN}$) computed using genotypes for all genotyped sows with phenotypic data born from cohort 1 to cohort 6, were used as covariates to estimate inbreeding depression.

## Results

### Range of values and interpretation of the genomic inbreeding coefficients

The inbreeding coefficients investigated differ in the range of values that they can contain and, with the exception of ${F}_{NEJ}$, their ranges depend on the allele frequency in the base population ${p}_{(0)}$. Coefficient ${F}_{NEJ}$ ranges from 0 to 1 because it is the proportion of homozygous SNPs. At the individual level, values for ${F}_{L\&H}$ range from − ∞ to 1, and those for ${F}_{VR1}$, ${F}_{VR2}$ and ${F}_{YAN}$ range from − 1 to ∞ (Figs. 3, 4, and 5, in Zhang et al. [27]). When all SNP genotypes are homozygous, ${F}_{L\&H}$ equals 1 and when all are heterozygous, it ranges from − ∞ to − 1. ${F}_{VR1}$ and ${F}_{VR2}$ cover the entire range (from − 1 to ∞) both when all SNP genotypes are homozygous or heterozygous. Finally, when all SNP genotypes are homozygous, ${F}_{YAN}$ ranges from 0 to ∞ and when they all are heterozygous, ${F}_{YAN}$ equals − 1. Thus, values for ${F}_{L\&H}$, ${F}_{VR1}$, ${F}_{VR2}$ and ${F}_{YAN}$ can be outside the permitted ranges for probabilities and correlations. Nevertheless, as inbreeding coefficients, they can still be interpreted as the proportional loss or gain in variability (heterozygosity) relative to the variability in the base population, with a negative value indicating that variability has been gained and a positive value that variability has been lost. It is also possible to gain more than 100% of the initial variability but it is not possible to lose more than 100%. A value equal to 1 indicates that all the variability that was present in the base population has been lost but a value greater than 1 indicates that more variability than what existed initially has been lost, which does not make sense.

### Expected values of genomic inbreeding coefficients based on the single locus model

**Fig. 1:**
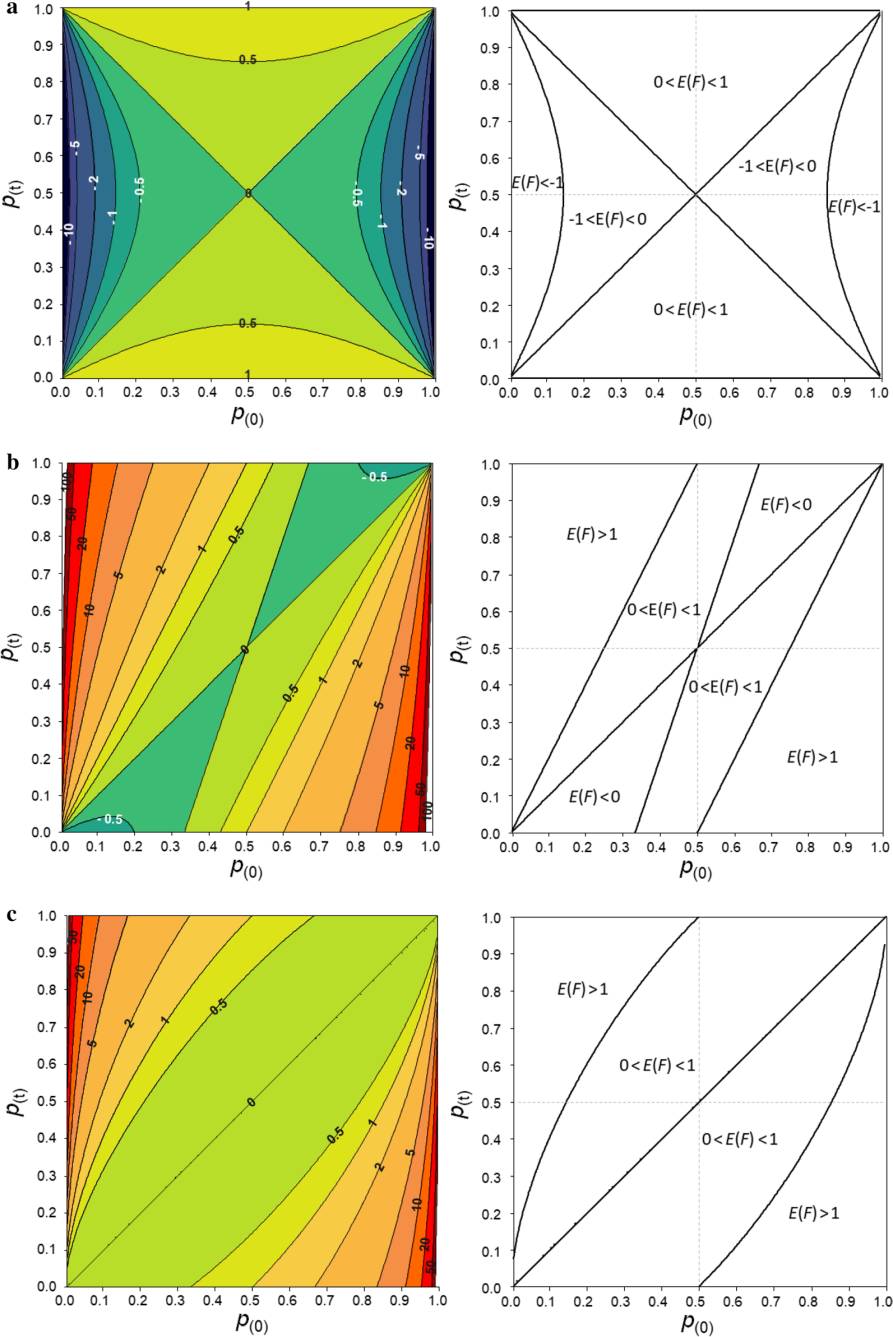
Expected inbreeding coefficient based on excess of homozygosity ($${F}_{L\&H}$$) (**a**) and expected inbreeding coefficients computed from the diagonal elements of the genomic relationship matrices of VanRaden (methods 1 and 2; $${F}_{VR}$$ = $${F}_{VR1}$$  = $${F}_{VR2}$$) (**b**) and of Yang ($${F}_{YAN})$$ (**c**) as a function of starting and current allele frequencies at a single locus. On the right, the grid of initial and current frequencies is divided in regions where the expected value of $$F$$ is < − 1, < 0, between − 1 and 0, between 0 and 1, or > 1

Expected values for ${F}_{L\&H}$, ${F}_{VR1}$ (or ${F}_{VR2}$) and ${F}_{YAN}$ based on the single locus model for the whole range of starting (${p}_{(0)}$) and current (${p}_{(t)}$) frequencies are shown in Fig. 1. Note that for a single locus model $E\left({F}_{VR1}\right)=E\left({F}_{VR2}\right)=E\left({F}_{VR}\right)$.

The expected value for ${F}_{L\&H}$ (Fig. 1a) ranged from − ∞ and 1. When the frequency of the minor allele increases (i.e. ${p}_{\left(t\right)}>{p}_{\left(0\right)}$) towards 0.5, ${E(F}_{L\&H})$ becomes negative, which indicates that some variability has been gained. This makes sense given that the maximum variability occurs when the frequency is 0.5. Given that the upper limit of ${E(F}_{L\&H})$ is 1, when using this coefficient, one never expects more variability to be lost than the variability that initially existed. ${E(F}_{L\&H})$ takes the value of 1 when the SNP becomes fixed, which is equivalent to all the variability being lost.

The expected value for ${F}_{VR}$ based on the diagonals of VanRaden’s GRM is within the range [0, 1] for some combinations of ${p}_{(0)}$ and ${p}_{(t)}$, but for many other combinations it is outside this range (Fig. 1b). In fact, $E({F}_{VR})$ ranges from − 1 to ∞. This means that $E({F}_{VR})$ can indicate that some variability has been gained but this gain can never be greater than 100% of the initial variability, as the lower limit is − 1. It also means that $E({F}_{VR})$ can indicate that more than 100% of the initial variability is lost, as it can take values higher than 1 (up to ∞).

In the right panel of Fig. 1b, the grid of initial and current frequencies is divided in regions where $E({F}_{VR})$ is < 0, between 0 and 1, or > 1. When the frequency of the minor allele is doubled (i.e. ${p}_{\left(t\right)}=2{p}_{\left(0\right)}$) but still lower than 0.5, $E\left({F}_{VR}\right)=1$, which means that 100% of the variability has been lost in the current generation when, in fact, variability has increased. For instance, if  ${p}_{(0)}$= 0.25 and  ${p}_{(t)}$= 0.5, $E\left({F}_{VR}\right)$ indicates that all the initial variability has been lost, although the maximum variability is reached at a frequency of 0.5. When the frequency of the minor allele more than doubles (i.e. ${p}_{\left(t\right)}>2{p}_{\left(0\right)}$), $E\left({F}_{VR}\right)$ becomes > 1 (for instance, for ${p}_{\left(0\right)}$ = 0.1 and ${p}_{\left(t\right)}$ = 0.3, $E\left({F}_{VR}\right)$ = 2.2), which indicates that more than 100% of the initial variability has been lost, which is unreasonable. When the initial frequency of the minor allele is lower than 0.33 and decreases, then $E\left({F}_{VR}\right)$ < 0, which indicates that variability has increased relative to its initial value. This is also the case when the minor allele is lost (${p}_{\left(t\right)}$ = 0). Thus, although variability in the current generation is lower than in the initial generation in these cases, $E\left({F}_{VR}\right)$ incorrectly indicates that some variability has been gained.

On the one hand, although for a particular individual in the population, ${F}_{YAN}$ can be negative (up to − 1), contrary to $E\left({F}_{VR}\right)$, $E\left({F}_{YAN}\right)$ is never smaller than 0 (it ranges from 0 to ∞; Fig. 1c), which indicates that the level of heterozygosity cannot become larger than the level that existed initially, which is unreasonable. On the other hand, and as for $E\left({F}_{VR}\right)$, $E\left({F}_{YAN}\right)$ can be greater than 1, implying that more heterozygosity than what existed initially can be lost. In addition, although increasing the frequency of the minor allele towards 0.5 increases variability, $E\left({F}_{YAN}\right)$ can indicate a decrease in variability. For instance, when ${p}_{(0)}$= 0.1 and remains at 0.1 in the current generation, $E\left({F}_{YAN}\right)$ = 0. However, if the frequency increases to 0.2, $E\left({F}_{YAN}\right)$ becomes greater than 0 ($E\left({F}_{YAN}\right)$ = 0.11), which indicates that some variability has been lost. And, if it increases to 0.5 (in theory a value at which the variability is maximum), $E\left({F}_{YAN}\right)$ becomes greater than 1 ($E\left({F}_{YAN}\right)$ = 1.78), which indicates that more than 100% of the initial variability was lost.

When the initial frequency (${p}_{(0)}$) is set to 0.5, the expected value for ${F}_{L\&H}$, ${F}_{VR}$ and ${F}_{YAN}$ is the same regardless of the current frequency (${p}_{(t)}$) (see Additional file 1: Figure S1). In this scenario, these three coefficients range from 0 (when ${p}_{(t)}$ remains at 0.5) to 1 (when the SNP becomes fixed; i.e. ${p}_{(t)}$ = 0 or 1).

**Fig. 2:**
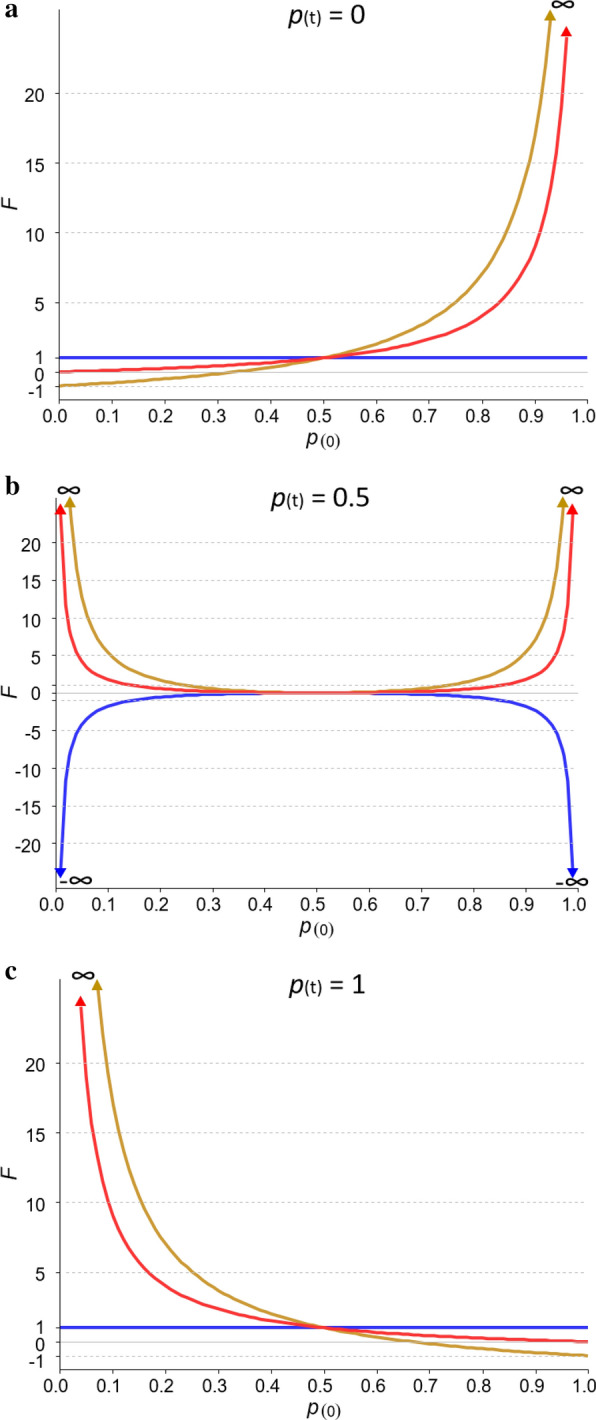
Expected ${F}_{L\&H}$, ${F}_{VR} ({F}_{VR1}={F}_{VR2})$ and ${F}_{YAN}$ in the generation in which the reference allele was lost (**a**), driven to a frequency of 0.5 (**b**), or fixed (**c**) relative to the initial frequency (${p}_{(0)}$). ${F}_{L\&H}$: blue line, ${F}_{VR}$: brown line, ${F}_{YAN}$: red line

Figure 2 shows the same profiles as in Fig. 1, but with the reference allele driven to a frequency of 0 (Fig. 2a), 0.5 (Fig. 2b) or 1 (Fig. 2c) in the current generation. Note that there is some redundancy in Fig. 2a, c since fixation of the major allele is equivalent to loss of the minor allele. For any value, ${E(F}_{L\&H})$ = 1 when the SNP becomes fixed (${p}_{(t)}$ = 0 or 1), regardless of the initial frequency, as expected (Fig. 2a, c). However, when the major allele is lost (Fig. 2a) or when the minor allele is fixed (Fig. 2c), both ${E(F}_{VR})$ and ${E(F}_{YAN})$ take values greater than 1 (in fact their upper limit is ∞). Losing the minor allele leads to negative values for ${E(F}_{VR})$ when ${p}_{(0)}$ < 1/3 and its limit is − 1 (Fig. 2a). Another way of looking at this is that fixing the major allele leads to negative values for ${E(F}_{VR})$ when ${p}_{(0)}$ > 2/3, and its limit is also − 1 (Fig. 2c). In these scenarios, the value of ${E(F}_{YAN})$ remains equal to 1. It is interesting to note that when ${p}_{(t)}$= 0.5, ${E(F}_{YAN})$, and to a lesser extent ${E(F}_{VR})$, behave as a mirror image of ${F}_{L\&H}$ (Fig. 2b).

In summary, expected values for ${F}_{L\&H}$, ${F}_{VR1}$, ${F}_{VR2}$ and ${F}_{YAN}$ depend on frequency changes. When the inbreeding coefficient is interpreted as an indicator of loss or gain of variability, ${F}_{L\&H}$ gives sensible values but ${F}_{VR1}$, ${F}_{VR2}$, and ${F}_{YAN}$ do not. In fact, $E({F}_{L\&H})$ follows the trend of loss or gain in heterozygosity due to changes in allele frequencies. When the minor allele frequency (MAF) decreases (i.e. when heterozygosity decreases relative to that in a reference base population), $E({F}_{L\&H})$ increases. However, $E({F}_{VR1})$ and $E({F}_{VR2})$ can lead us to think that: (i) more than 100% of the initial variability is lost; and, even worse, (ii) variability has increased when in reality it has decreased or vice versa. $E({F}_{YAN})$ also leads to inconsistent results since it never indicates that variability has increased, but it can indicate that more than 100% of the initial variability is lost.

### Patterns of genomic inbreeding in the population of Guadyerbas pigs

Summary statistics for the different inbreeding coefficients, computed both at the individual level and at the regional (window) level, are in Table 3 for the first and last cohorts. Average values for each coefficient at the individual and regional levels were practically the same but those at the regional level varied much more than those at the individual level, particularly for cohort 6. The proportion of homozygous loci (${F}_{NEJ}$) increased by 5% from cohort 1 to cohort 6. Coefficient ${F}_{NEJ}$ had a much higher average and a lower standard deviation than the other coefficients. Coefficients that are weighted by the initial frequencies (i.e. ${F}_{L\&H}$, ${F}_{VR1}$, ${F}_{VR2}$ and ${F}_{YAN}$) were on average less than 0 for cohort 1 (about − 0.1) and became positive (up to ~ 0.2) for cohort 6.

**Table 3:** Mean, standard deviation (SD) and minimum and maximum values for the different genomic inbreeding coefficients when computed at the individual animal or genomic region level in cohorts 1 and 6 of the Guadyerbas population

Cohort		Individual level				Regional level			
		Mean	SD	Min	Max	Mean	SD	Min	Max
1	${F}_{NEJ}$	0.616	0.024	0.580	0.674	0.615	0.084	0.300	0.968
	${F}_{L\&H}$	− 0.095	0.070	− 0.198	0.071	− 0.095	0.122	− 0.517	0.373
	${F}_{VR1}$	− 0.095	0.076	− 0.230	0.119	− 0.095	0.122	− 0.517	0.372
	${F}_{VR2}$	− 0.088	0.108	− 0.279	0.211	− 0.088	0.106	− 0.450	0.340
	${F}_{YAN}$	− 0.088	0.053	− 0.165	0.061	− 0.088	0.106	− 0.450	0.341
6	${F}_{NEJ}$	0.669	0.025	0.631	0.743	0.669	0.124	0.307	1.000
	${F}_{L\&H}$	0.056	0.070	− 0.052	0.268	0.056	0.353	− 2.939	1.000
	${F}_{VR1}$	0.120	0.079	− 0.014	0.417	0.111	0.352	− 0.853	2.717
	${F}_{VR2}$	0.175	0.108	0.023	0.609	0.172	0.558	− 0.876	4.118
	${F}_{YAN}$	0.090	0.076	− 0.014	0.364	0.089	0.215	− 0.465	1.306

**Fig. 3:**
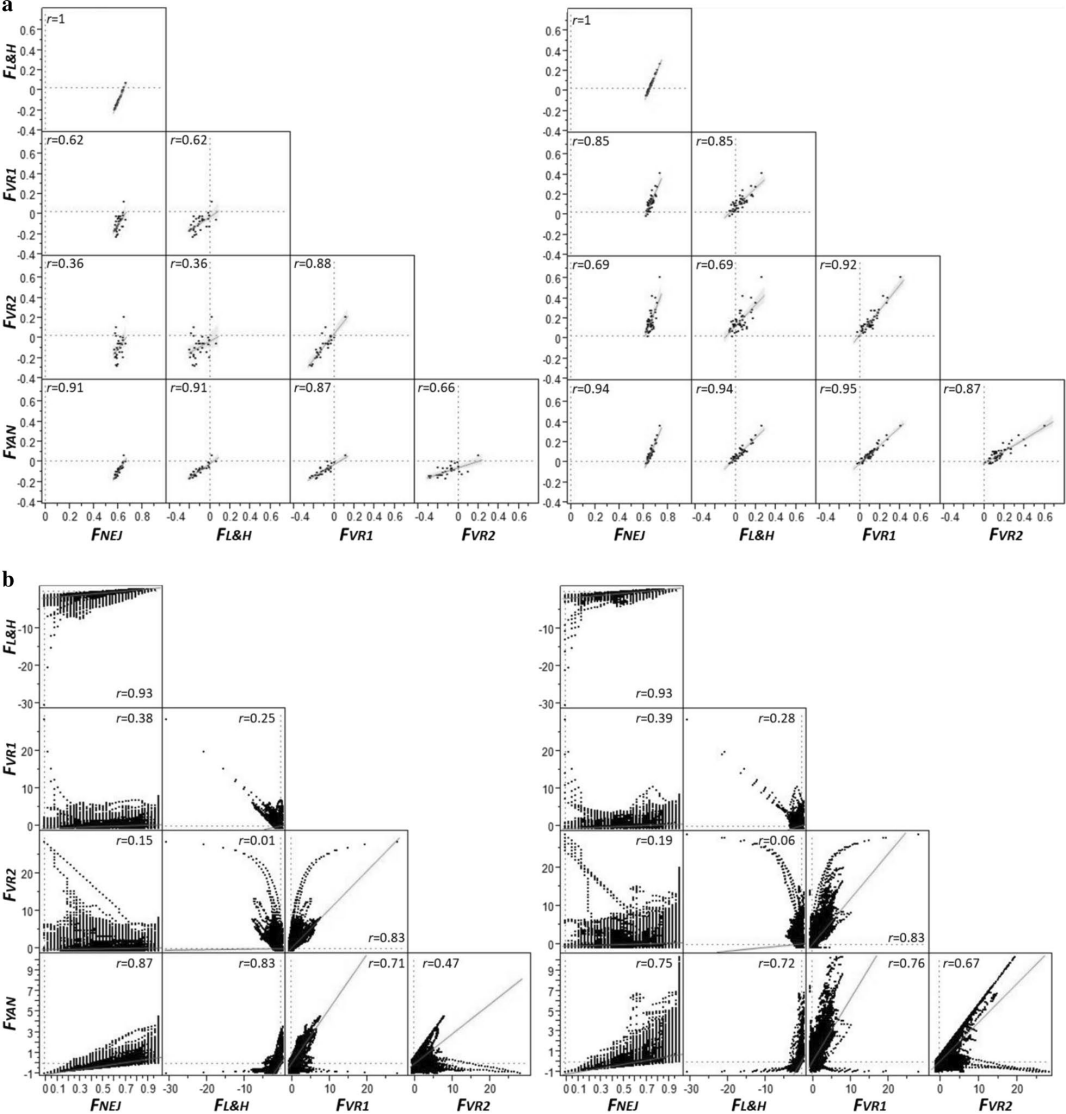
Scatter plots for inbreeding coefficients ${F}_{NEJ}$, ${F}_{L\&H}$, ${F}_{VR1}$, ${F}_{VR2}$ and ${F}_{YAN}$ in the Guadyerbas population when computed at the individual animal (**a**) or genomic region (**b**) level in cohorts 1 (left panels) and 6 (right panels), and the corresponding correlation coefficients ($r$)

Pairwise correlations between coefficients computed both at the individual and regional (window) level are in Fig. 3. Correlations at the individual animal level (which are averages across the genome) ranged from 0.4 to 1 for cohort 1 and from 0.7 to 1 for cohort 6. As expected, the correlation between ${F}_{NEJ}$ and ${F}_{L\&H}$ was 1. Correlations higher than 0.9 were also found between ${F}_{YAN}$ and ${F}_{NEJ}$, ${F}_{YAN}$ and ${F}_{L\&H}$, ${F}_{YAN}$ and ${F}_{VR1}$, and ${F}_{VR1}$ and ${F}_{VR2}$. The lowest correlations were between ${F}_{VR2}$ and ${F}_{NEJ}$ and between ${F}_{VR2}$ and ${F}_{L\&H}$, but these correlations increased from ~ 0.4 for cohort 1 to ~ 0.7 for cohort 6, which could be due to the loss of rare alleles over time but also to random fluctuations. At the regional genomic level, changes in frequencies can be more exaggerated, which results in lower correlations between coefficients than at the individual animal level, particularly for those involving VanRaden’s coefficients.

**Fig. 4:**
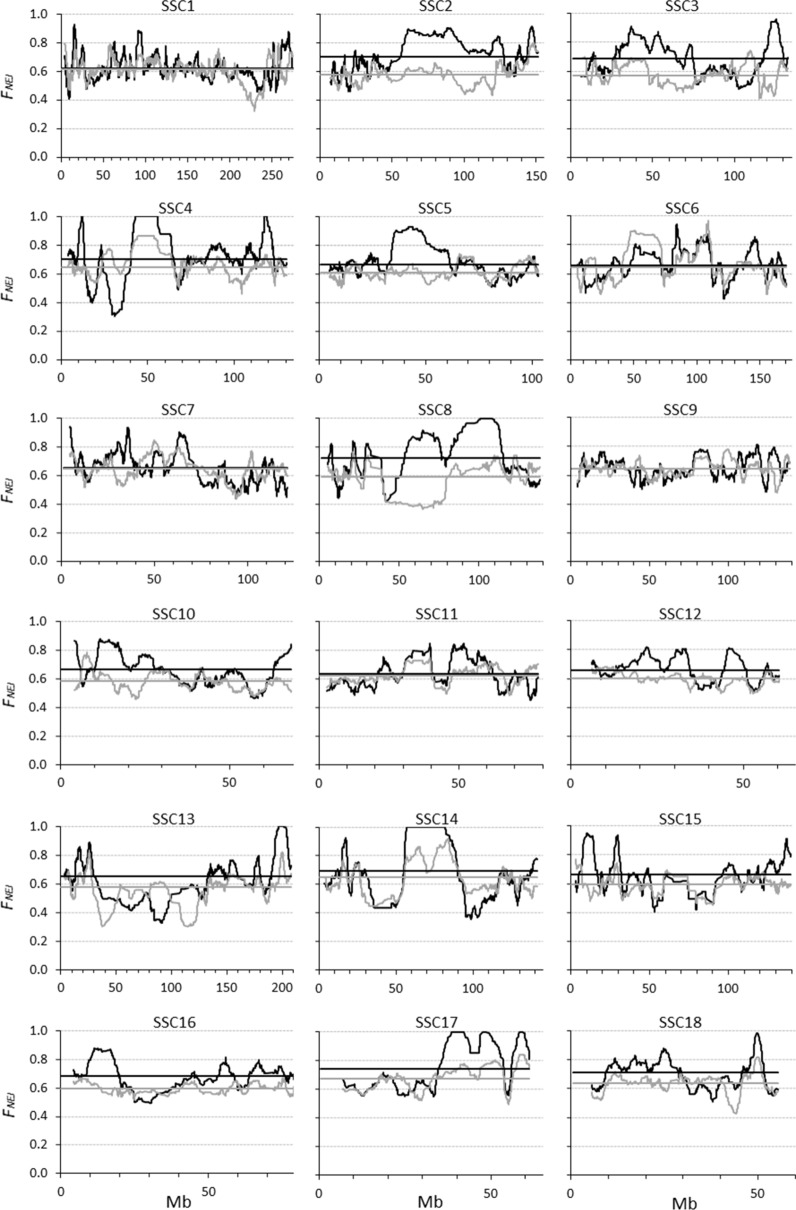
Evolution of the proportion of the genome that becomes homozygous (i.e. *F*_*NEJ*_) from cohort 1 (grey lines) to cohort 6 (black lines) for the different chromosomes (SSC) in the Guadyerbas population when using SNPs with non-zero minor allele frequencies. The horizontal lines represent averages across the genome

The pattern of homozygosity clearly varied across chromosomes and across regions within chromosomes (Fig. 4). For several genomic regions, SNPs that were still segregating in cohort 1 became fixed in cohort 6 (see for example, *Sus scrofa* (SSC) chromosomes 4, 8, 13, 14 and 17).

**Fig. 5:**
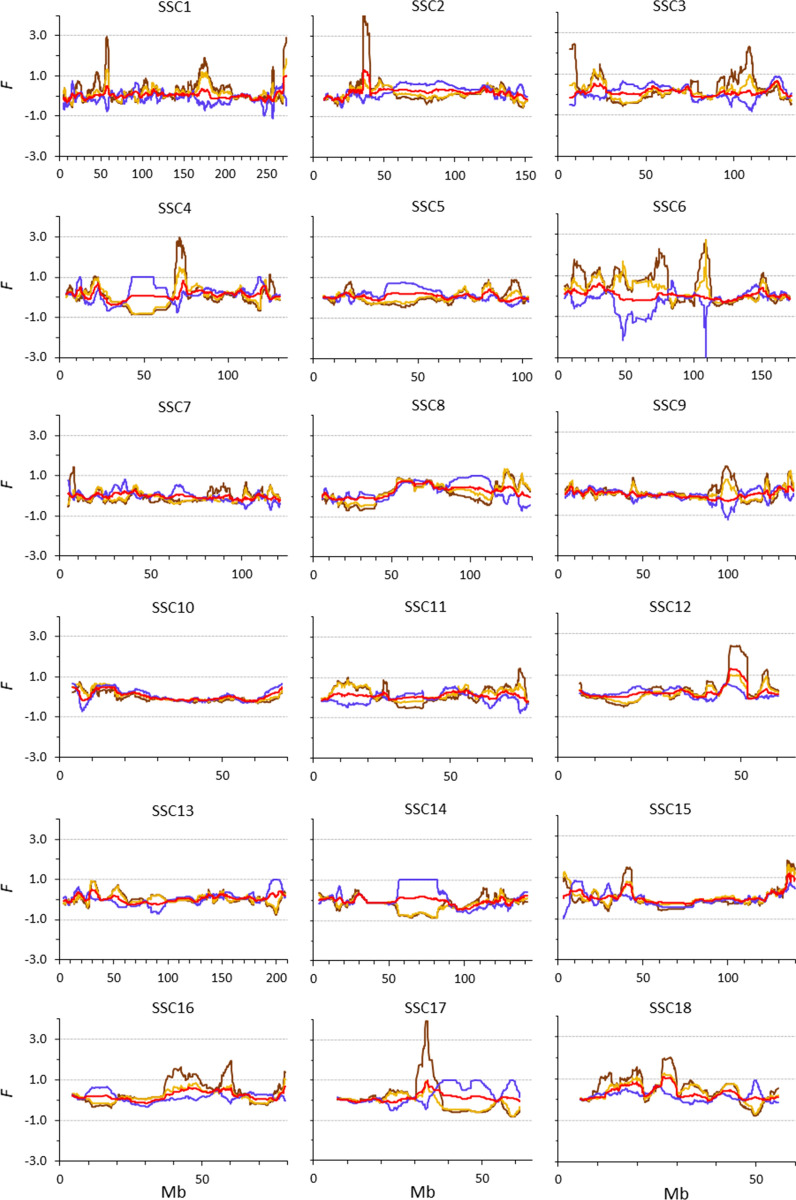
Patterns of different measures of genomic inbreeding (${F}_{L\&H}$ blue line, ${F}_{VR1}$ light brown line, ${F}_{VR2}$ dark brown line, ${F}_{YAN}$ red line) in cohort 6 for different chromosomes (SSC) in the Guadyerbas population when using SNPs with non-zero minor allele frequencies in cohort 1

Figure 5 compares the patterns of the different coefficients across the genome for cohort 6. Here, we only consider the SNPs that segregated in cohort 1. In general, the patterns differed a lot between coefficients. It is interesting to note that, in general, the patterns for ${F}_{VR1}$ and ${F}_{VR2}$ were mirror images of those for ${F}_{L\&H}$. One particularly striking result is that in regions where SNPs had become fixed (see also Fig. 4), ${F}_{L\&H}$ was equal to 1 whereas ${F}_{VR1}$ and ${F}_{VR2}$ were negative with large absolute values. Two very clear examples are the region between 43 and 56 Mb on SSC4 and the region between 58 and 82 Mb on SSC14. In both these regions, the initial frequency of the minor allele was very low (${p}_{(0)}$ ≤ 0.1), and the allele was already lost in cohort 6 (${p}_{(t)}$ = 0). At all positions within these regions, ${F}_{L\&H}$ was equal to 1, while ${F}_{VR1}$ and ${F}_{VR2}$ became negative (about − 0.8 in the SSC4 region and ranging from − 0.9 to − 0.6 in the SSC14 region), which incorrectly suggests that some variability was gained, and ${F}_{YAN}$ was low (about 0.1 in the SSC4 region and ranging from 0.1 to 0.2 in the SSC14 region). These observations agree with the expectations described above and lead us to conclude that ${F}_{L\&H}$ is a much more valuable measure of change in variability than ${F}_{VR1}$ or ${F}_{VR2}$. For the regions where all variability was lost, ${F}_{L\&H}$ is expected to indicate that this is the case, but both ${F}_{VR1}$ and ${F}_{VR2}$ indicate that variability was gained.

In addition, there are some regions for which the variability increased from cohort 1 to cohort 6, as ${F}_{NEJ}$ was lower in the latter (Fig. 4), e.g. the regions between 102 and 112 Mb on SSC3, between 41 and 72 Mb on SSC6, and between 81 and 97 Mb on SSC13. In all these cases, ${F}_{L\&H}$ did indeed show this increase in variability since it became negative. However, ${F}_{VR1}$ and ${F}_{VR2}$ were again like mirror images of ${F}_{L\&H}$, while ${F}_{YAN}$ was positive but close to 0. These observations also agree with expectations. For instance, the average ${p}_{(0)}$ and ${p}_{(t)}$ in the SSC13 region were 0.31 and 0.40, respectively. With this change in frequency, $E({F}_{L\&H})$ varied from − 1 to 0, while the expected values for ${F}_{VR1}$, ${F}_{VR2}$ and ${F}_{YAN}$ were all between 0 and 1. Also remarkable are the high peaks observed for ${F}_{VR2}$. In some regions on SSC2 and SSC17, ${F}_{VR2}$ reached a value as high as 4. In these regions (between 35 and 38 Mb on SSC2 and between 33 and 34 Mb on SSC17), there are SNPs with rare alleles (${p}_{(0)}$ < 0.1) which had a high increase in frequency (${p}_{(t)}$ > 0.3), and under these circumstances, ${F}_{VR2}$ is expected to reach very high positive values (Fig. 1b), while ${F}_{L\&H}$ is expected to become negative (Fig. 1a).

With VanRaden's and Yang’s coefficients, and in particular ${F}_{VR2}$, a higher inbreeding coefficient is assigned to an individual that is homozygous for a rare allele than to an individual that is homozygous for a common allele. Thus, ${F}_{VR1}$, ${F}_{VR2}$, and ${F}_{YAN}$ put a greater weight on SNPs that have a low MAF. Based on this, in addition to the scenario considered so far, in which all the SNPs segregating in cohort 1 (MAF > 0) were used to calculate the inbreeding coefficients, we analyzed two additional scenarios with different MAF thresholds in cohort 1: (i) using only the common variants (here defined as SNPs with MAF > 0.05); and (ii) using only the very common variants (here defined as SNPs with MAF > 0.25). This allowed us to determine how the differences between coefficients were affected by MAF.

**Fig. 6:**
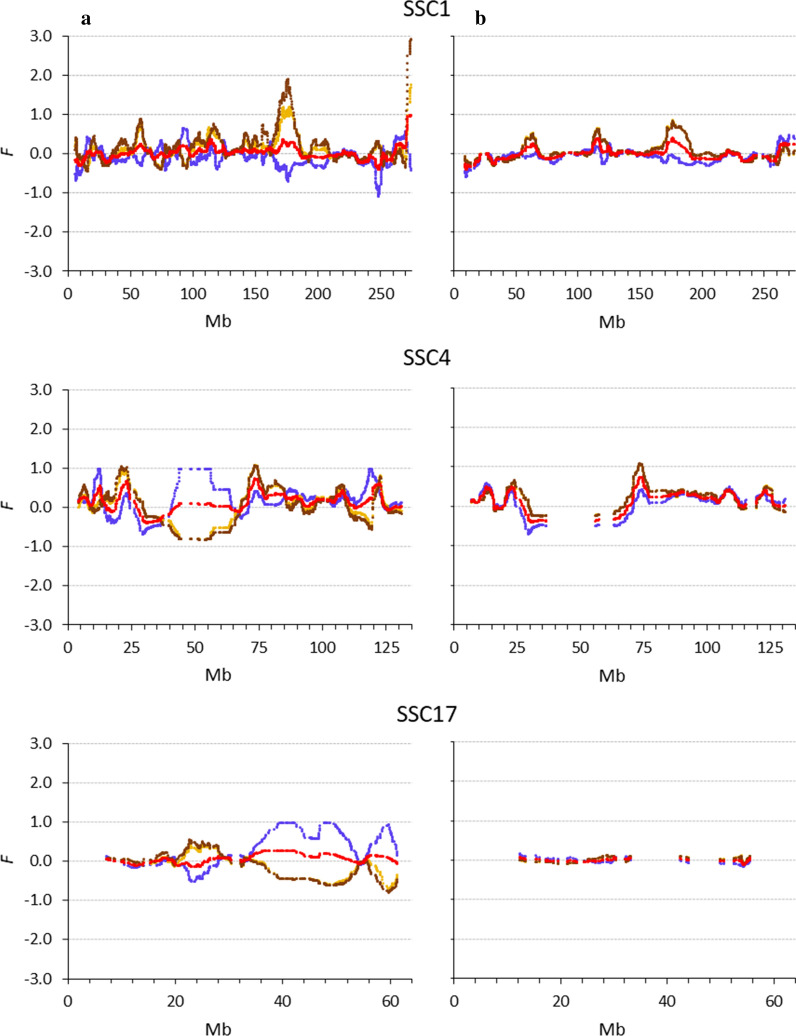
Patterns of different measures of genomic inbreeding (${F}_{L\&H}$ blue line, ${F}_{VR1}$ light brown line, ${F}_{VR2}$ dark brown line, ${F}_{YAN}$ red line) in cohort 6 for chromosomes 1, 4, and 17 in the Guadyerbas population when using SNPs with minor allele frequencies > 0.05 (**a**) or > 0;25 (**b**) in cohort 1

Figure 6 shows the patterns of each coefficient computed using only SNPs with a MAF > 0.05 or > 0.25 for three chromosomes. When only SNPs with a MAF higher than 0.05 in cohort 1 were used, some of the strong peaks previously obtained disappeared, in particular for ${F}_{VR2}$ (Fig. 6 versus Fig. 5). Using an even stricter MAF filter (MAF > 0.25) led to very similar patterns for all inbreeding coefficients (Fig. 6). In fact, pairwise correlations between coefficients increased considerably compared to those shown in Fig. 3. When only SNPs with a MAF higher than 0.25 were used, all correlations were higher than 0.95, both in cohorts 1 and 6. SNPs with a MAF higher than 0.05 and higher than 0.25 represented 92% (16,532 SNPs) and 54% (9,716 SNPs), respectively, of the total number of segregating SNPs in cohort 1. Note that SNP density greatly decreased in some regions when SNPs were filtered on MAF, resulting in the discontinuities seen in Fig. 6. These results show that the inconsistencies described earlier for ${F}_{VR1}$, ${F}_{VR2}$, and ${F}_{YAN}$ occur when there are SNPs with a low MAF, and in practice such SNPs exist. Removing loci with rare alleles would defeat the rationale behind the coefficients that intentionally give more weight to rare alleles.

#### Consequences for the estimation of inbreeding depression

**Fig. 7:**
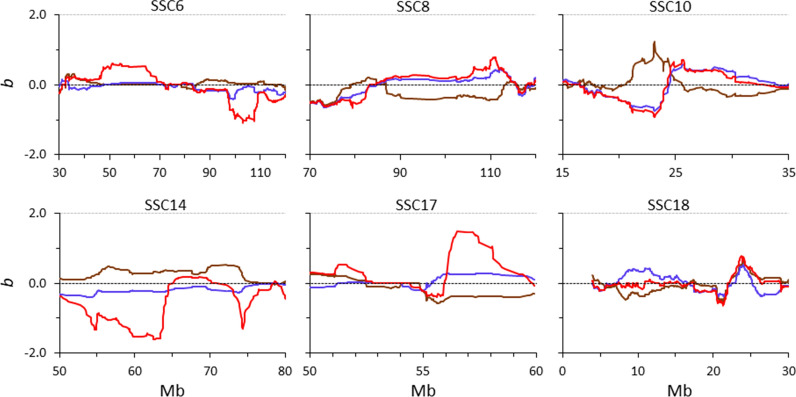
Patterns of the rate of inbreeding depression (*b*) for number of piglets born alive in the Guadyerbas population when computed using different measures of genomic inbreeding (${F}_{L\&H}$ blue line, ${F}_{VR2}$ brown line, ${F}_{YAN}$ red line) for specific regions of six chromosomes. All genotyped sows with phenotypic data that were born from cohort 1 to cohort 6 were included in the analyses

For each of the three measures of $F$, the patterns of the rate of inbreeding depression (i.e. the regression coefficient, *b*) for all chromosomes are shown in Additional file 2: Figure S2. Across the whole genome, the estimates of *b* differed substantially between the methods used to compute $F$. In some regions within chromosomes, estimates of $b$ were very similar across methods but in other regions they differed greatly, not only in magnitude but also in sign. As an illustration, Fig. 7 shows selected regions within chromosomes for which the conclusions on the magnitude and sign of the rate of inbreeding depression differ substantially. In the regions from 50 to 70 Mb and from 98 and 109 Mb on SSC6, estimates of $b$ were close to 0 when using ${F}_{L\&H}$ and ${F}_{VR2}$ but clearly different from 0 when using ${F}_{YAN}$. However, in other regions (e.g. from 90 to 113 Mb on SSC8, from 20 to 24 Mb on SSC10, from 50 to 65 Mb on SSC14, and from 56 to 60 Mb on SSC17), ${F}_{L\&H}$ and ${F}_{YAN}$ led to estimates of $b$ that were of the same sign but opposite to estimates obtained when using ${F}_{VR2}$. In the region from 7.5 to 11 Mb on SSC18, the sign of the estimate of $b$ obtained with ${F}_{L\&H}$ was opposite to that obtained with ${F}_{VR2}$ and ${F}_{YAN}$.

Pairwise correlations between estimates of rates of inbreeding depression computed with ${F}_{L\&H}$, ${F}_{VR2}$, and ${F}_{YAN}$ are in Additional file 3: Figure S3. Across the genome, correlations involving estimates based on ${F}_{VR2}$ ranged from ~ 0.4 to 0.5, whereas the correlation between estimates based on ${F}_{L\&H}$ and ${F}_{YAN}$ was high (0.84). About 40% of the estimates of $b$ in Additional file 3: Figure S3 were of opposite sign when based on ${F}_{L\&H}$ and ${F}_{VR2}$, and this percentage decreased to ~ 27% when estimates were based on ${F}_{VR2}$ and ${F}_{YAN}$ and to ~ 15% when using ${F}_{L\&H}$ and ${F}_{YAN}$. This reinforces the idea that care should be taken when interpreting estimates of inbreeding depression obtained with different measures of genomic inbreeding.

## Discussion

The inbreeding coefficient has been defined as a probability [9] or as a correlation [8], and thus its legitimate range is between 0 and 1 or between − 1 and 1, respectively. Another interpretation of the inbreeding coefficient, which we have used here, is in terms of loss or gain of variability relative to a reference base population. Under this interpretation, on the one hand, a negative value (even a value lower than − 1) makes sense and means that some variability has been gained. On the other hand, a value higher than 1 means that more variability than that initially existing has been lost, which is not reasonable.

Using a single locus model, we provided expectations for different genomic inbreeding coefficients that have been widely used in the literature. These expectations help to understand the patterns of these coefficients when they are computed using thousands of SNPs in a real population. Except for ${F}_{NEJ}$, none of the genomic coefficients considered here (i.e. those depending on allele frequencies) match with Malécot’s or Wright’s definition of the inbreeding coefficient as a probability or correlation, respectively, since their values can be outside the legitimate ranges [47]. In fact, at the individual animal level, ${F}_{L\&H}$ can range from − ∞ to 1 and ${F}_{VR1}$, ${F}_{VR2}$, and ${F}_{YAN}$ from − 1 to ∞. At the population level (see the section on expected genomic coefficients under a single locus model above), the ranges are the same as at the individual level, except for ${F}_{YAN}$, which can range from 0 to ∞. When these coefficients are interpreted as indicators of whether variability is gained or lost over generations, ${F}_{L\&H}$ leads to sensible results but ${F}_{VR1}$, ${F}_{VR2}$, and ${F}_{YAN}$ do not. This also has consequences when estimating the rate of inbreeding depression for specific genome regions since different measures of inbreeding can lead to very different results.

Although ${F}_{L\&H}$ is not a probability or a correlation, this measure of inbreeding is useful for determining whether variability is lost or gained. The largest variability (heterozygosity) for biallelic loci occurs at allele frequencies equal to 0.5. When a rare allele increases its frequency towards 0.5, ${F}_{L\&H}$ indicates that variability is gained, as expected. In addition, this measure of inbreeding never indicates that more variability than what existed in the initial generation was lost. In contrast, ${F}_{VR1}$, ${F}_{VR2}$, and ${F}_{L\&H}$ also do not match with a definition of inbreeding based on the proportion of variability lost or gained. In fact, for some ${p}_{(0)}$ and ${p}_{(t)}$ combinations, these three coefficients can indicate that variability is lost when the MAF increased towards 0.5, and this loss can be even higher than 100% of the initial variability. Moreover, ${F}_{VR1}$ and ${F}_{VR2}$ can take values that indicate that heterozygosity in the current generation is higher than what existed in the initial generation, although some heterozygosity has actually been lost. This does not occur with ${F}_{L\&H}$ at the population level since ${E(F}_{L\&H})$ is never negative, i.e. it always indicates that heterozygosity decreases although, in theory, it could increase.

One of the advantages of using genomic rather than pedigree data to measure inbreeding is the possibility of investigating the pattern of inbreeding along the genome. Here, we compared the patterns of each genomic coefficient computed from thousands of SNPs obtained with the Illumina PorcineSNP60 BeadChip v1 in a population of Iberian pigs. This population is highly inbred, with an estimated effective population size as low as 20 [54, 55]. The behaviour of each inbreeding coefficient observed with real data was well explained by the expectations developed. In the six generations (i.e. from cohort 1 to cohort 6), many SNPs became fixed (see Fig. 4). This loss of variability was captured by ${F}_{NEJ}$ (which is simply the proportion of homozygous SNPs) and was also clearly reflected in the value for ${F}_{L\&H}$ (${F}_{L\&H}$ = 1). However, the negative values obtained for ${F}_{VR1}$ and ${F}_{VR2}$ for these regions indicate that variability in cohort 6 was higher than in cohort 1. For regions where the variability increased from cohort 1 to cohort 6, ${F}_{L\&H}$ had negative values (which reflects reality), while ${F}_{VR1}$, ${F}_{VR2}$, and ${F}_{YAN}$ had positive values (which do not reflect the reality). Although ${E(F}_{YAN})$ predicts that variability can be gained in some circumstances, this occurs when, in fact, variability has been lost. In general, ${F}_{VR1}$ and ${F}_{VR2}$ behave similarly, although values for ${F}_{VR2}$ are more extreme. Values for ${F}_{YAN}$ lie between those for ${F}_{L\&H}$ and those for ${F}_{VR1}$ and ${F}_{VR2}$, and generally, are close to 0. Given these results, it is clear that, when specific genome regions are targeted to control inbreeding, the choice of the genomic coefficient used should be done with care.

We have analysed the behaviour of five genomic measures of inbreeding that have been widely used in the literature. However, other measures have been proposed (see review by Kardos et al. [32]). For instance, both the PLINK [56] and GCTA [18] software provide a modification of ${F}_{L\&H}$ (their ${F}^{II}$, here referred to as ${F}_{L\&H2}$). Although to our knowledge, ${F}_{L\&H2}$ is not widely used, it is interesting to note that the difference between ${F}_{L\&H}$ and ${F}_{L\&H2}$ is equivalent to the difference between ${F}_{VR1}$ and ${F}_{VR2}$ in that it only differs in how the summation over SNPs is carried out. This is clearly illustrated by the patterns of these coefficients obtained for the Guadyerbas pig population (see Additional file 4: Figure S4). The patterns for ${F}_{L\&H}$ were, in general, mirror images of the patterns for ${F}_{VR1}$ and those for ${F}_{L\&H2}$ were, in general, mirror images of patterns for ${F}_{VR2}$. Another widely used measure of genomic inbreeding, which we have not considered here, is the coefficient ${F}_{ROH}$ based on continuous runs of homozygosity (ROH) [11, 12]. Contrary to the coefficients considered here, which are computed on a SNP-by-SNP basis, ${F}_{ROH}$ is computed on a segment basis and has the advantages that (i) its values range from 0 to 1 (${F}_{ROH}$ is the proportion of the genome that is in ROH); and (ii) it can distinguish between distant (based on short ROH) and recent (based on long ROH) inbreeding. Its ability to detect inbreeding depression has been proven in multiple studies [3, 11, 12, 19, 21, 25, 26, 28, 32, 36, 39, 40, 42, 57]. However, the exact definition of ${F}_{ROH}$ varies across studies, depending on the choice of the parameters to define a ROH (e.g. number of heterozygous genotypes permitted in a ROH, minimum SNP density required, maximum distance allowed between two consecutive homozygous SNPs, and minimum number of SNPs). Because of this, population-wide expected values for ${F}_{ROH}$ are difficult to derive.

The pedigree-based numerator relationship matrix, NRM [58] has been used very extensively for many years to estimate the genetic covariance between individuals that are genetically evaluated via best linear unbiased prediction (BLUP). With the advent of genomic evaluations [59], the NRM has been replaced by more precise realised relationship matrices, which has led to an increase in the accuracy of predicted breeding values (e.g. [60]). Replacing NRM with GRM has also led to two other applications. The first application was the object of our study. Given that self-relationships in the NRM are expected to be equal to 1 plus the individual’s inbreeding coefficient, genomic inbreeding coefficients have been also obtained from the diagonals of the GRM. However, as we have shown here, this is not always justified. In the ideal situation, with an infinite number of independent loci and absence of migration, mutation, and selection, the average allele frequencies remain constant over generations and all measures, except ${F}_{NEJ}$, are expected to produce unbiased estimates of the inbreeding coefficient (IBD) relative to a base population that is in Hardy–Weinberg equilibrium [42, 61]. However, in more realistic situations, the proposed genomic estimators of inbreeding can result in very different outcomes. The second application of the GRM is based on the fact that the NRM is equal to twice the matrix of coancestry coefficients and, as such, it has been used to optimize contributions of breeding candidates by applying the optimal contribution method (OC) for maintaining genetic variability and avoiding inbreeding depression in genetic conservation programs [47, 62, 63]. In this context, GRM have been used in OC, replacing NRM. de Cara et al. [64] and Gomez-Romano et al. [65] showed that the use of the coancestry matrix computed from Nejati-Javaremi’s GRM in OC resulted in a higher level of genetic diversity (measured as expected heterozygosity) being maintained than when using the NRM in OC. Morales-González et al. [47] showed that the amount of genetic variability retained was higher when using Nejati-Javaremi’s or Li and Horvitz’s matrices in OC than when using VanRaden and Yang’s GRM, although the latter were also efficient in controlling the loss of genetic diversity. Thus, in the context of optimizing contributions for maintaining diversity, VanRaden and Yang’s GRM are useful. In fact, it has been suggested [66] that although the use of VanRaden and Yang’s GRM in OC results in less variability being maintained, they could lead to allele frequencies that are closer to those in the original population (i.e. allele frequencies would tend to be unchanged), which can be an objective in conservation programs, particularly in ex situ conservation programs, where the final aim is reintroduction to the wild [67].

It has been suggested that the use of whole-genome sequence data could produce improved genomic inbreeding coefficient estimates [23] because it captures the many variants with rare alleles, which may not be included in the SNP panels due to their ascertainment bias. However, including a higher proportion of variants with rare alleles is expected to lead to even more inconsistent results than those shown here when using ${F}_{YAN}$ and ${F}_{VR1}$, and particularly ${F}_{VR2}$.

Under the infinitesimal model, the NRM is a matrix of covariances of breeding values but, importantly, it is also twice the matrix of coancestry coefficients, with self-coancestries on the diagonal. Given that the relationship between self-coancestry ($f$) and inbreeding ($F$) coefficients is $f=0.5(1+F)$, the NRM provides estimates of $F$. GRM are also covariance matrices that have proven to work very well in genomic predictions. However, although ${F}_{NEJ}$ and ${F}_{L\&H}$ correctly indicate when variability is lost or gained, this is not the case with ${F}_{VR1}$, ${F}_{YVR2}$, and ${F}_{YAN}$.

## Conclusions

Except for ${F}_{NEJ}$ (which ranges from 0 to 1), values for the genomic coefficients investigated here are outside the ranges of Malécot’s and Wright’s definitions of coefficient of inbreeding. When using a third interpretation of inbreeding in terms of loss or gain of variability, ${F}_{L\&H}$ gives sensible values but ${F}_{VR1}$, ${F}_{VR2}$, and ${F}_{YAN}$ do not. In fact, the expectations derived here at the population level show some inconsistencies for these three coefficients. These include indications that (i) more variability than what initially existed can be lost (${F}_{VR1}$, ${F}_{VR2}$, and ${F}_{YAN}$); (ii) variability has decreased when in reality it has increased (${F}_{VR1}$, ${F}_{VR2}$, and ${F}_{YAN}$); (iii) variability has increased when in reality it has decreased (${F}_{VR1}$ and ${F}_{VR2}$); and (iv) it is not possible to gain more variability than what existed initially (${F}_{YAN}$). The expectations developed here clearly explain the different patterns of these coefficients obtained for a highly inbred pig population when using thousands of SNP genotypes.

## Supplementary Information

**Additional file 1: Figure S1.** Expected ${F}_{L\&H}$, ${F}_{VR}({F}_{VR1}={F}_{VR2})$ and ${F}_{YAN}$ at a given current frequency (${p}_{(t)})$ when the initial frequency of the reference allele (${p}_{(0)}$) is set to 0.5. ${F}_{L\&H}$: blue line, ${F}_{VR}$: brown line, and ${F}_{YAN}$: red line.

**Additional file 2: Figure S2.** Patterns of the rate of inbreeding depression for number of piglets born alive ($b$) when computed using different measures of genomic inbreeding (${F}_{L\&H}$: blue line, ${F}_{VR2}$: brown line, and ${F}_{YAN}$: red line) for each chromosome of the Guadyerbas genome. All genotyped sows with phenotypic data that were born from cohort 1 to cohort 6 were included in the analyses.

**Additional file 3: Figure S3.** Scatter plots for rates of inbreeding depression for number of piglets born alive ($b$) when computed using ${F}_{L\&H}$, ${F}_{VR2}$ or ${F}_{YAN}$ against each other, and corresponding correlation coefficients (*r*). All genotyped sows with phenotypic data that were born from cohort 1 to cohort 6 were included in the analyses. Values indicated with different colors correspond to regions presented in Fig. 7.

**Additional file 4: Figure S4.** Patterns of different measures of genomic inbreeding (${F}_{L\&H}$: blue line, ${F}_{L\&H2}$: grey line, ${F}_{VR1}$: light brown line, ${F}_{VR2}$: dark brown line, and ${F}_{YAN}$: red line) in cohort 6 for each chromosome of the Guadyerbas genome when using SNPs with a MAF higher than 0 in cohort 1.

## Data Availability

The datasets analyzed during the current study are available from the corresponding author on reasonable request.
