# Supplementary figures and images for: The value of genomic relationship matrices to estimate levels of inbreeding
Source: Genet Sel Evol. 2021 May 1;53:42. doi: 10.1186/s12711-021-00635-0 (PMC8088726; doi:10.1186/s12711-021-00635-0)

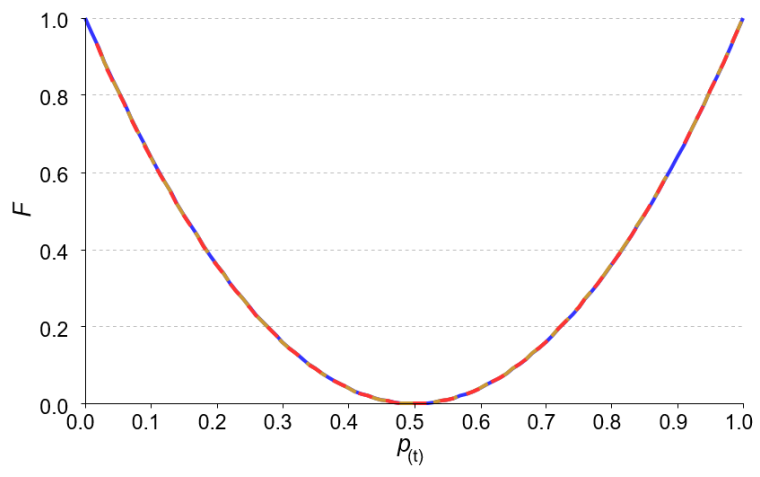

Supplement: Supplementary file 1 — Additional file 1: Figure S1. Expected ${F}_{L\&H}$FL&H, ${F}_{VR}({F}_{VR1}={F}_{VR2})$FVR(FVR1=FVR2) and ${F}_{YAN}$FYAN at a given current frequency (${p}_{(t)})$p(t)) when the initial frequency of the reference allele (${p}_{(0)}$p(0)) is set to 0.5. ${F}_{L\&H}$FL&H: blue line, ${F}_{VR}$ [file 12711_2021_635_MOESM1_ESM.pdf]

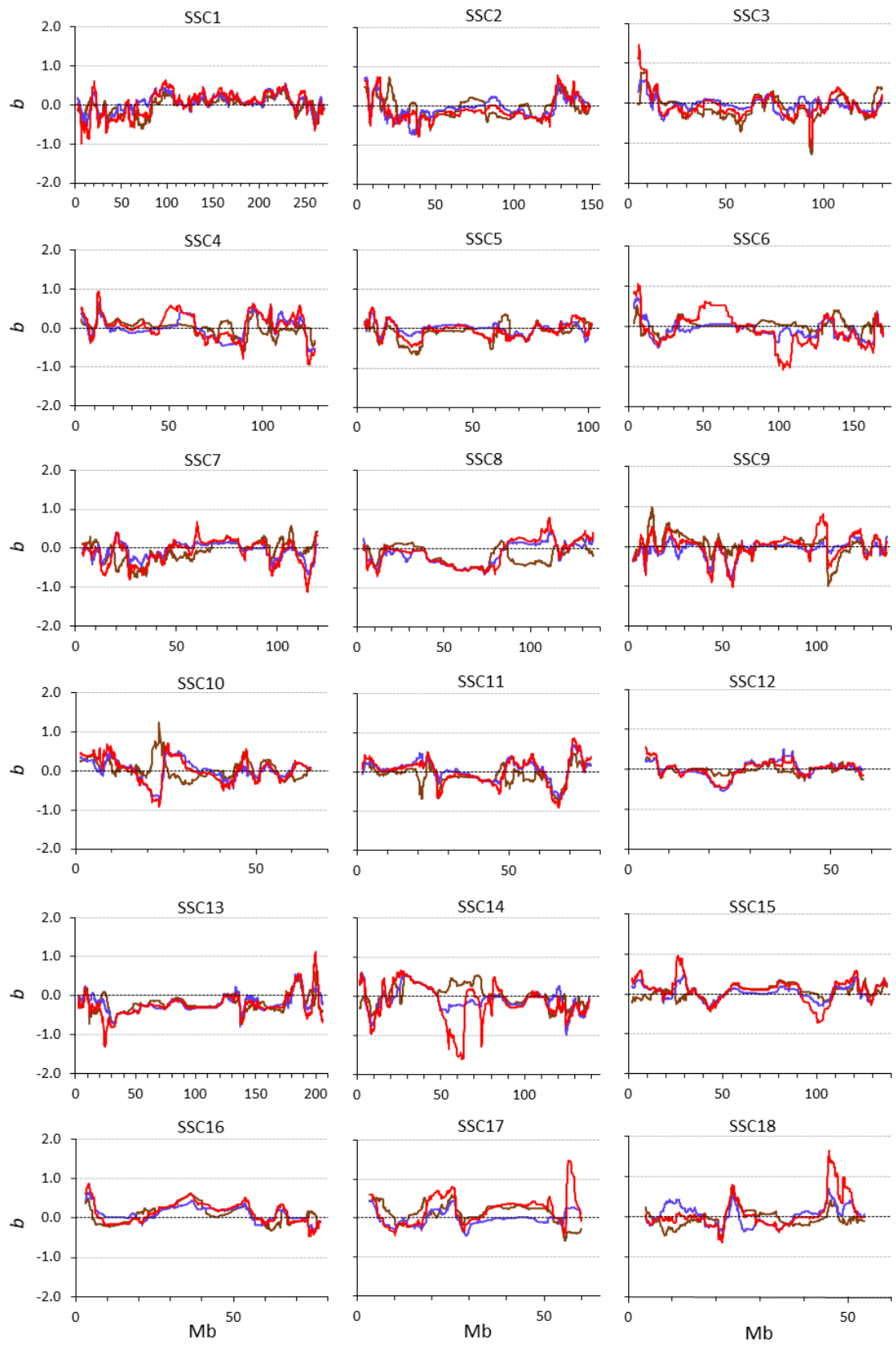

Supplement: Supplementary file 2 — Additional file 2: Figure S2. Patterns of the rate of inbreeding depression for number of piglets born alive ($b$b) when computed using different measures of genomic inbreeding (${F}_{L\&H}$FL&H: blue line, ${F}_{VR2}$FVR2: brown line, and ${F}_{YAN}$FYAN: red line) for each chromosome of the Guadyerbas genome. All genotyped sows with phenotypic data that were born from cohort 1 to cohort 6 were included in the analyses. [file 12711_2021_635_MOESM2_ESM.pdf]

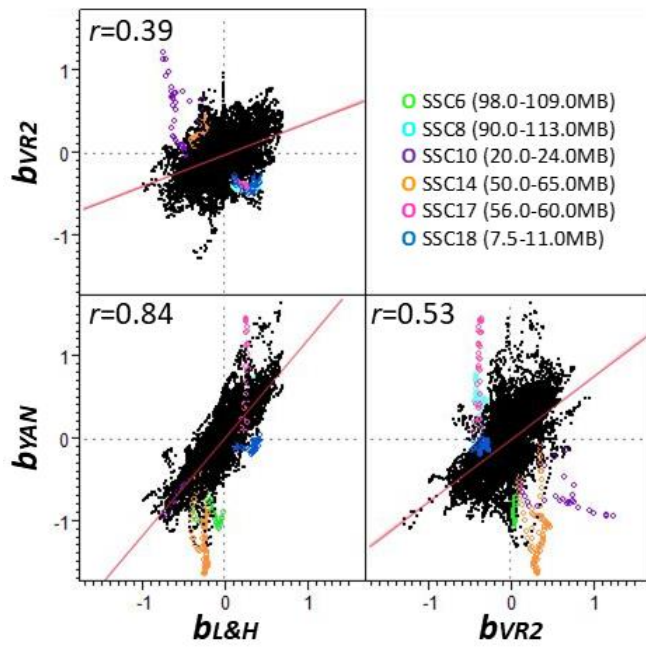

Supplement: Supplementary file 3 — Additional file 3: Figure S3. Scatter plots for rates of inbreeding depression for number of piglets born alive ($b$b) when computed using ${F}_{L\&H}$FL&H, ${F}_{VR2}$FVR2 or ${F}_{YAN}$FYAN against each other, and corresponding correlation coefficients (r). All genotyped sows with phenotypic data that were born from cohort 1 to cohort 6 were included in the analyses. Values indicated with different colors correspond to regions presented in Fig. 7. [file 12711_2021_635_MOESM3_ESM.pdf]

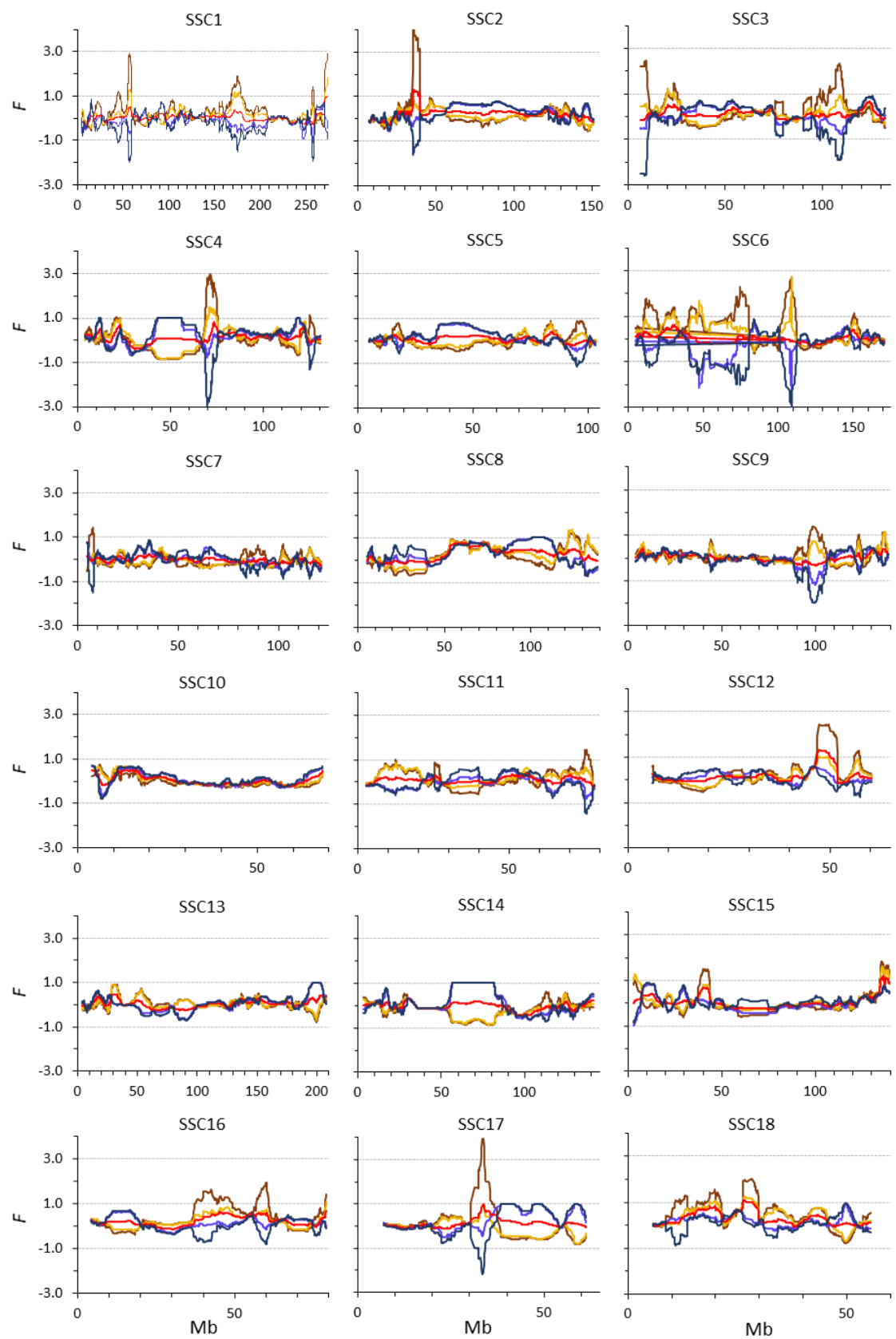

Supplement: Supplementary file 4 — Additional file 4: Figure S4. Patterns of different measures of genomic inbreeding (${F}_{L\&H}$FL&H: blue line, ${F}_{L\&H2}$FL&H2: grey line, ${F}_{VR1}$FVR1: light brown line, ${F}_{VR2}$FVR2: dark brown line, and ${F}_{YAN}$FYAN: red line) in cohort 6 for each chromosome of the Guadyerbas genome when using SNPs with a MAF higher than 0 in cohort 1. [file 12711_2021_635_MOESM4_ESM.pdf]
